# The Use of Artificial Intelligence in Writing Scientific Review Articles

**DOI:** 10.1007/s11914-023-00852-0

**Published:** 2024-01-16

**Authors:** Melissa A. Kacena, Lilian I. Plotkin, Jill C. Fehrenbacher

**Affiliations:** 1 Department of Orthopaedic Surgery, Indiana University School of Medicine, Indianapolis, IN 46202 USA; 2 Department of Anatomy, Cell Biology & Physiology, Indiana University School of Medicine, Indianapolis, IN 46202 USA; 3 Indiana Center for Musculoskeletal Health, Indiana University School of Medicine, Indianapolis, IN 46202 USA; 4 Richard L. Roudebush VA Medical Center, Indianapolis, IN 46202 USA; 5 Department of Pharmacology and Toxicology, Indiana University School of Medicine, Indianapolis, IN 46202 USA; 6 Stark Neuroscience Research Institute, Indiana University School of Medicine, Indianapolis, IN 46202 USA

**Keywords:** Artificial intelligence (AI), ChatGPT, Scientific writing, Osteoporosis, Musculoskeletal system, Fracture healing, Neural regulation, Alzheimer's disease, COVID-19, SARS-CoV-2

## Abstract

**Purpose of Review:**

With the recent explosion in the use of artificial intelligence (AI) and specifically ChatGPT, we sought to determine whether ChatGPT could be used to assist in writing credible, peer-reviewed, scientific review articles. We also sought to assess, in a scientific study, the advantages and limitations of using ChatGPT for this purpose. To accomplish this, 3 topics of importance in musculoskeletal research were selected: (1) the intersection of Alzheimer’s disease and bone; (2) the neural regulation of fracture healing; and (3) COVID-19 and musculoskeletal health. For each of these topics, 3 approaches to write manuscript drafts were undertaken: (1) human only; (2) ChatGPT only (AI-only); and (3) combination approach of #1 and #2 (AI-assisted). Articles were extensively fact checked and edited to ensure scientific quality, resulting in final manuscripts that were significantly different from the original drafts. Numerous parameters were measured throughout the process to quantitate advantages and disadvantages of approaches.

**Recent Findings:**

Overall, use of AI decreased the time spent to write the review article, but required more extensive fact checking. With the AI-only approach, up to 70% of the references cited were found to be inaccurate. Interestingly, the AI-assisted approach resulted in the highest similarity indices suggesting a higher likelihood of plagiarism. Finally, although the technology is rapidly changing, at the time of study, ChatGPT 4.0 had a cutoff date of September 2021 rendering identification of recent articles impossible. Therefore, all literature published past the cutoff date was manually provided to ChatGPT, rendering approaches #2 and #3 identical for contemporary citations. As a result, for the COVID-19 and musculoskeletal health topic, approach #2 was abandoned midstream due to the extensive overlap with approach #3.

**Summary:**

The main objective of this scientific study was to see whether AI could be used in a scientifically appropriate manner to improve the scientific writing process. Indeed, AI reduced the time for writing but had significant inaccuracies. The latter necessitates that AI cannot currently be used alone but could be used with careful oversight by humans to assist in writing scientific review articles.

## Introduction

Time is valuable, and advancements of artificial intelligence (AI) provide new avenues to save this precious resource. Although AI language models have been in development for years, understanding of their potential and use by the general population increased dramatically with the introduction of ChatGPT by OpenAI in November of 2022. Generative pretrained transformer (GPT) is a technology that utilizes the branch of computer science known as natural language processing (NLP) to establish communication between computers and humans [1]. NLP allows the software to understand and generate human language, and the program has been fed a massive amount of text in its training. This body of information is combined with neural network programming to create a large language model (LLM) such as ChatGPT that can communicate with humans by predicting appropriate text responses based upon input training data [2]. The capabilities and applications of AI are numerous, and it can perform tasks in seconds that would take most human users significantly more time and effort. For this reason, AI has begun to inch its way into many different fields such as medicine and research [3, 4].

Recently, there has been much discussion in the research community on the use of AI in scientific writing. Some contend that AI is a useful aid in writing, but many scientists and publishers reject the use of AI to write papers single-handedly or listing an LLM as the author of a paper [5–8]. However, it is undeniable that AI can assist in scholarly writing, as ChatGPT, Google Bard, Bing, and other LLMs are skilled language programs that can assist with grammar, vocabulary, and writing style. Beyond the LLMs, other AI resources can be used to perform plagiarism checks and serve as search engines to mine available literature databases for information and resources, essential tools for a researcher writing a manuscript. Therefore, use of AI could help save time for all writers, especially those with the additional challenge of writing in a non-native language [3]. Despite all of these benefits, using AI in scientific writing requires scrutiny and skepticism. There have been notable instances where the misuse of AI, such as with the use of ChatGPT, has led to serious consequences. One occurrence resulted in the fining of lawyers who referred to fictitious court citations generated by ChatGPT [9]. Further, there are other limitations to the use of AI, as AI can infringe upon copyright laws, experiences “artificial hallucinations,” produces inaccurate or biased results, and cannot weigh the importance of various specific sources when answering questions. Artificial hallucinations are instances of AI text generation, containing falsified information, that AI attempts to confidently pass off as true based upon the common knowledge of a topic [10]. These identified limitations have led to concern that, as AI is more widely adopted by scientists, use of AI will facilitate manuscripts of low quality that contain falsified information. It has already begun to fool some human reviewers by writing believable abstracts [11]. If AI can write believable abstracts, is it currently capable of writing a publishable full-length scientific review?

Scientific review articles provide readers with a succinct summary as well as a synthesis of the existing studies, observations, and gaps in knowledge for a particular area of research. Even though they are a useful tool for researchers to learn more about different areas of research, scientific reviews are time- and labor-intensive to produce. They require extensive literature review, a well-structured text that accurately summarizes the scientific findings, and a commentary on the gaps in the knowledge and what can be done to address them, as well as easy to understand illustrations for the benefit of the reader to quickly understand the relationships and interactions among key points from the text. AI has the potential to offset some of these limitations. AI can search the internet and analyze potential sources much faster than a human [12]. Also, AI language models specialize in text creation and can generate sentences about a certain topic that flow smoothly and are easy to understand. Therefore, AI may be able to write a quality, full-length review article in less time than a human alone. Another option for writing a scientific review article would be to do a full-length literature review by hand and then provide AI with that specific list of references to write a review article. This may reduce or eliminate the hallucination of information by AI. Furthermore, providing AI with references would manually increase the validity of the writing by considering the reputation of the sources, the relevance to the topic at hand, the inclusion of contemporary findings within the field of research, and the inclusion of findings that otherwise would not be accessible to AI (journal paywalls). This option mostly tests AI’s capabilities of information extraction and language development to write a quality review article.

**Fig. 1:**
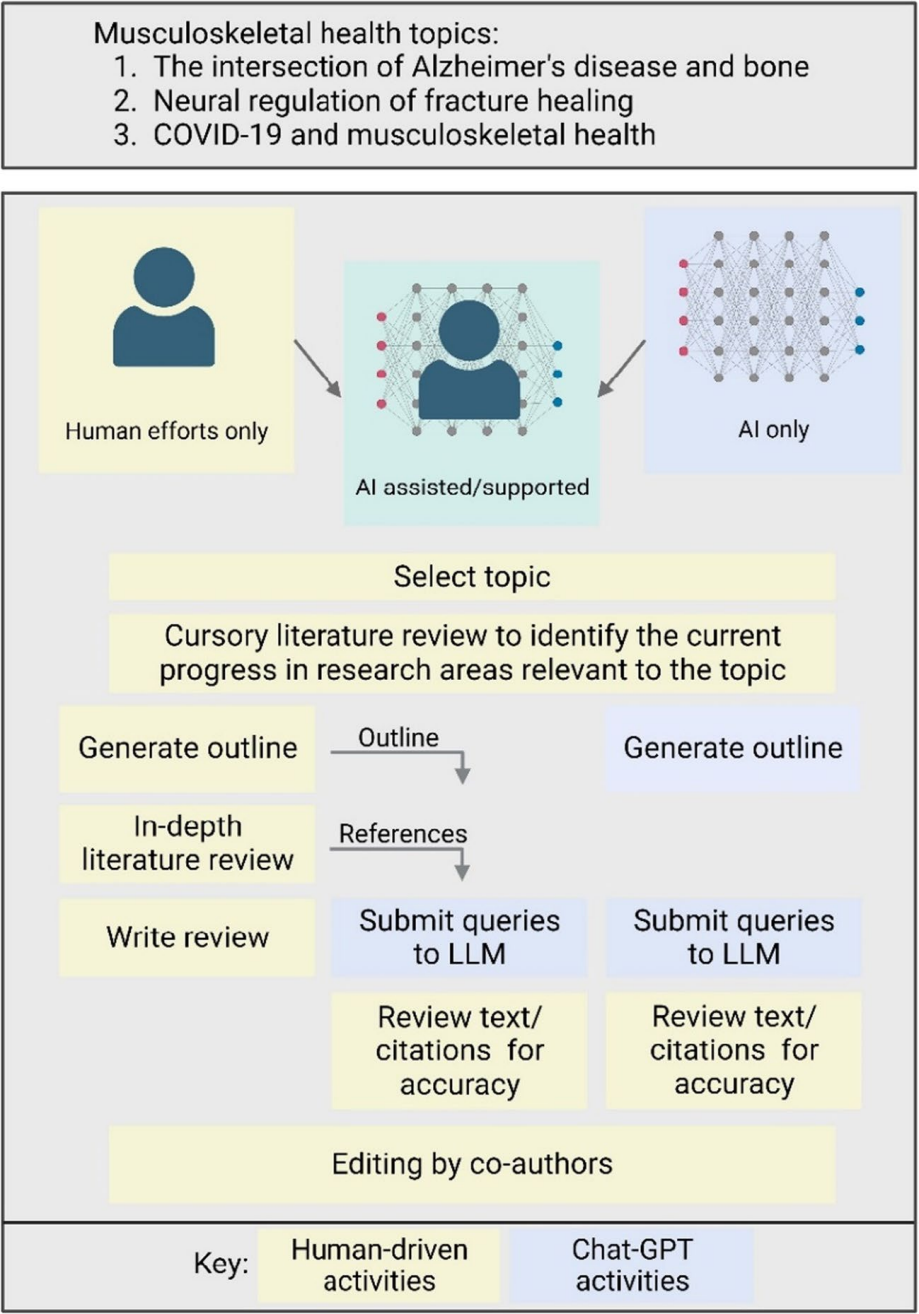
Overview of experimental study design employed to examine the utility of using ChatGPT to write scientific review articles about the intersection of Alzheimer’s disease and bone, neural regulation of fracture healing, and COVID-19 and musculoskeletal health. Three approaches were taken for each scientific topic: human only (yellow), AI only (blue), and a combined approach — AI assisted (green). A number of outcomes were measured for each approach during the course of the study

To address the original question of AI’s ability to write a publishable quality, full-length scientific review, we compared three writing strategies and evaluated whether AI utilization can save time in the composition of a scientific review article. As shown in Fig. 1, the writing strategies included (1) the traditional process of completing a literature review, outlining the manuscript, and writing the review article (human efforts only or “human”); (2) a process using AI only to complete the literature review, outline the manuscript, and write the first draft of the review article (AI-only or “AIO”); and (3) a process in which the literature review and outline were generated by humans (taken from #1 above, the human process), but AI was used to write the first draft of the review article (AI-assisted/supported or “AIA”). These three writing strategies were then used to write review articles on 3 different topics of interest in the musculoskeletal field: (1) the intersection of Alzheimer’s disease and bone [13–15]; (2) neural regulation of fracture healing [16–18]; and (3) COVID-19 and musculoskeletal health [19, 20]. With this background, we hypothesized that the human paper would take the most time and require the fewest changes between the first and final drafts. We also hypothesized that the AI-only paper would require the most changes but would take the least amount of time. Finally, we hypothesized that the AI-assisted paper would take an intermediate amount of time and require fewer changes than the AI-only paper.

## Methods

### Human-Generated and Written Review Articles

The traditional methods of writing a review article were employed for the human-generated writing style [13, 16, 19]. Specifically, a comprehensive literature review was completed, and an outline of relevant topics was created to help guide the authors in organizing and focusing their review article. Once a complete first draft was written, the manuscript underwent extensive editing and fact checking by all co-authors. Please see the associated Comment for each of the review articles to see the respective first drafts [21–23]. Reference citations were inserted into the manuscript using EndNote. A graphical abstract was created on BioRender.com. The idea of the graphical abstract was conceived by the primary author with input from other co-authors. This article was written and edited entirely by humans.

### AI-Only (AIO) Review Articles

An author without an extensive AI background began by experimenting to determine the best queries for generating their AI-only review articles [14, 17]. Please refer to the Comments for each review topic [21–23] for a complete listing of all queries used to generate the AIO review articles. The AI model that was used to write first drafts of papers was the ChatGPT Plus version using the GPT-4 language model (OpenAI). Of note, the April 2023–August 2023 version of ChatGPT was utilized for research and text generation. At that time, the knowledge cutoff for ChatGPT was September 2021. As a result, all articles identified during the generation of the human manuscript after this date were uploaded such that ChatGPT could access them. This was accomplished by using the “AskYourPDF” plugin feature that can only be accessed through the paid version of GPT-4 (i.e., not available with the free GPT-3.5 version). Importantly, as most of the COVID-19 and musculoskeletal health articles were published after September 2021, the resulting AIO manuscript essentially became an AI-assisted manuscript (see below) and therefore was abandoned.

The first step of the AI-only paper was to generate an outline and a title for the review article. While each set of authors (for the 3 different review topics) used different query strategies [21–23], an example from the “Neural regulation of fracture healing” topic is provided below:

You are a PhD-level biological researcher who has experience writing sophisticated research paper outlines. Write a 2.5-page outline for a paper on the neural regulation of fracture healing. Include one section on an introduction, additional sections on each of the major concepts that will be included in the paper (generate these topics by synthesizing the research conducted to study the various aspects of the nervous system that regulate fracture healing), and one section on the conclusion. Create more detailed, specific sub-sections for all of the sections except for the introduction and conclusion. Include bullet points under each section with the key, detailed facts that will be expanded upon in that section. Each specific, detailed fact should be written as a complete sentence. For the introduction and conclusion, include multiple bullet points (each one specific sentence) that lay out the content of those sections. Based on the outline, generate a witty but informative title for the research paper and include the title at the beginning of the outline.

After some minor edits were made, originally by querying GPT and then eventually by making human edits, this outline was fed back into ChatGPT, and each section of the paper was written using variations on the following query:

Next, use the outline to write Heading 5. Ensure it is at least 300 words in length. Write at the level of a biological researcher and include all citations from the primary articles where you obtain the information in the section. When citing conclusions made by primary sources, expand upon the experiments researchers completed to come to these conclusions. It is imperative that you are very specific. Ensure this section is clear, logical, and flows well.

Due to GPT’s character limit (4096 characters), which restricted the entire paper from being written in a single prompt, new prompts were used to generate each section/subheading and the sections were merged to generate the full manuscript. A new “chat session” was started each time a new section/subheading of the paper was written. Next, each citation generated by GPT was fact checked and replaced by the authors when the citation did not exist or when it did not match the content of the sentence. This was done to ensure that the final version of the manuscript was accurate and suitable for publication and would not mislead readers. Rewrites were completed using ChatGPT, but some human intervention was warranted. Reference citations were inserted into the manuscript using EndNote. Of note, the unedited, first drafts of all articles are provided in the Comment associated with each review topic [21–23].

A graphical abstract was attempted using OpenAI’s DALL-E program; however, the quality of the images produced was not publishable. Given this, the idea for a graphical abstract was generated by ChatGPT based on its analysis of the paper’s finalized abstract, and the graphical abstract was created by the authors on BioRender.com (the same process was used for the AI-assisted paper detailed below). Similarly, an attempt was made to query GPT to select which important, recently published sources to annotate, a requirement for publication in *Current Osteoporosis Reports*. However, due to GPT’s inability to access knowledge after September 2021, it was ultimately decided that the authors would select which articles to annotate based upon general guidance provided by GPT, but to have ChatGPT write the highlight related to the identified reference. A few tips provided by GPT were to annotate sources whose content was specific to the topic of the neural regulation of fracture healing, and to look for sources that had been cited by other papers and that were published in higher impact journals.

### AI-Assisted (AIA) Review Articles

For the AI-assisted review articles [15, 18, 20], ChatGPT-4 was used as outlined in the AI-only section above with the following differences. The AI-assisted article utilized the human-generated outline and all the references utilized to generate this article were provided using the AskYourPDF plugin as described above. Specifically, AskYourPDF was used to generate unique codes recognized by ChatGPT which corresponded to each PDF. This enabled the articles to be uploaded for analysis by ChatGPT, so this plugin was vital for this paper to be written. Through trial and error, it was found that ChatGPT was unable to properly analyze multiple codes in a single text box. Each code had to be uploaded to ChatGPT in a separate chat for proper analysis to occur. Slight differences in queries were utilized between authors but the general system described below was used. Again, please see the Comment associated with each review topic to see a complete listing of queries used [21–23].

**Query 1:** I need help writing a subheading of a review article about **(paper topic)**. The subheading I need help writing is **(subheading topic)**. Can I provide you with 10 documents using AskYourPDF that we can use to synthesize this section?

**Query 2:** Okay, I am going to upload each ID separately so that you may better process the information. After each ID, you may write a short summary of the key findings of that document. After all documents are uploaded, I will ask you to write the review. Are you ready or do you have any questions?

**> Proceed to upload each document ID in separate text boxes.**

**Query 3:** Okay that was the last one. I have now provided you with 10 documents (Document 1, Document 2, Document 3, Document 4, Document 5, Document 6, Document 7, Document 8, Document 9, Document 10). Please write an in-depth review of the linkage between **(paper topic)**. It’s okay if your discussion contains information outside of **(paper topic)**, only use these directions as a framework. Write at the level of a doctorate researcher. Use in-text citations when necessary. If there are multiple documents that contain a piece of information, use multiple citations at the end of a sentence.

**Query 4:** That is perfect! Please condense this review into approximately 300 words while retaining citations from all **(number)** documents. You do not need to include an introduction or conclusion to this section, focus only on the findings of the documents.

This system was repeated multiple times until the first draft of the paper was created. Reference citations were inserted into the manuscript using EndNote. As with all manuscripts, the paper went through rounds of fact checking and editing by all co-authors. Rewrites were completed using ChatGPT, but some human intervention was required. For the AI-assisted review articles, graphical abstracts were created by humans on BioRender.com, but the concept was provided by ChatGPT based on its analysis of the finished manuscript. Annotated references were those identified during the human-generated review, but ChatGPT generated the statement of significance.

### All Papers

A number of parameters were measured and compared between the 3 (or 2 for COVID-19) types of review articles, and other parameters were compared between the first draft and the final draft. The findings for each of these assessments are located in the Comment for each topic [21–23].

Assessments included tracking time spent during different stages/activities of the review writing process. This was tracked using the “Toggl” application. Activities were divided into preparation, literature review, writing (which included writing queries for ChatGPT), fact checking, editing, other, and total time spent. Preparation refers to time spent reading articles, watching videos, and experimenting with AI before beginning official query generation. “Other” tracks activities that do not fall into defined categories, such as graphical abstract creation and reference annotation. The time spent was also attributed to trainees (in the first 3 author positions of all review articles) versus faculty (positions 4 through last author).

Similarity scores between original and final drafts were calculated using software from CopyLeaks. These scores were tabulated for all papers to measure edits and changes implemented from the original to the final draft. The final draft was also examined for plagiarism similarity index scores using Turnitin software. This program compares the provided text to internet sources, academic journals, and previously submitted papers to determine a percentage of the text that is highly similar to outside sources.

During the fact checking process, the validity of the references was examined. References were flagged as incorrect if there was any error in the actual citation such as incorrect year, authors, title, and journal. References were also deemed incorrect if the text for which the citation was listed was not relevant to the reference. Further, references were marked as incorrect if they were fabricated. Additionally, the number of queries used for each step of the process was tracked. It should be noted that even the purchased version of ChatGPT limited one to 25 queries/3 h.

## Main Results and Conclusions

Specific results and conclusions found for each of the three scientific topic areas are discussed in their associated Comments [21–23]. For this mini study, we intentionally selected 3 musculoskeletal review topics to give a sample size of 3. We believe this was important and found some differences, even among our 3 topics. One example was the significant limitation of not being able to access the most recent findings available on the internet. This limitation impacted very recent areas of study, like COVID-19, more than the other topics of research; however, recently released LLMs are allowed to access the internet and use of these resources will likely overcome this limitation. Another difference between the 3 topics was the time spent during the revision stage. All review articles were peer-reviewed as per standard *Current Osteoporosis Reports* procedure. Six of the eight articles were returned with relatively minor revisions. Two of the eight articles were returned with the need for extensive reorganization of the manuscript text. Both of these articles were in the “Neural regulation of fracture healing” topic and happened to be the human- and AI-assisted articles, which both used the human-generated outline. Based on separate reviewer feedback, the order of the human-generated outline was more confusing than the AI-generated outline, suggesting an important advantage of using AI.

There were also many similarities between our groups. For example, the AI-only process was the fastest but had the most errors in the references. The numbers of inaccuracies in the AI-only group were high (up to 70% incorrect references). Left unchecked by those knowledgeable in the field, these references would have misinformed readers, which is not acceptable. Indeed, our findings are consistent with another report, whereby they tested the ability of ChatGPT to write research protocols, which found that 61% of cited references were accurate, 23% existed but were improperly cited, and 16% were completely fabricated and a product of AI hallucination [24].

The first draft following the AI-assisted process resulted in a higher percentage of plagiarism compared to the human only or AI-only drafts. Although not specifically quantified, qualitative comments from faculty were that AI writing was easier to read than the human-generated manuscripts. This was likely owing to AI writing like one is taught in school in a prescriptive paragraph format: tell them what you are going to tell them, tell them, and then tell them what you told them. AI does repeat itself numerous times, for example it will use words like “multifaceted” and “complex” numerous times throughout a document. Another observation related to a limitation of AI is that it cannot always synthesize the information provided to make meaningful connections between concepts, as we expect human writers to do. Again, this limitation will likely be minimized with time as AI will learn with iterative processing, but currently this is a struggle where faculty needed to provide more significant editing to connect the ideas in several cases.

One finding that stands out is that, in our Alzheimer’s disease and bone AI-only review, the total time was significantly lower than for all other papers. We suspect this may be an unintentional consequence of the more advanced training of the first author (a research assistant professor). This research faculty member had participated in writing grants on this topic area and may have been able to guide ChatGPT or the editing process more efficiently than those with less knowledge on the topic. For all other papers, the first authors were a postdoctoral fellow (*n* = 1), graduate students (*n* = 1), or medical students between their first and second year of medical school (*n* = 5).

AI is likely here to stay, thus exploring its utility in scientific writing is timely. As with every new technology, there are pros and cons. Figuring out how to expand the pros while limiting the cons is critical to successful implementation and adoption of the technology. Here, and even more so in our companion Comments for each topic, we provide the reader with our queries so that our experiences can help others to improve the generation of queries that can assist in efficient utilization of ChatGPT-4. As with most technologies, by the time this is published, there will be new advances, but it can still provide insight and even a blueprint for novices beginning to use AI in their scientific writing applications.

## Data Availability

Data will be made available upon reasonable request.
